# Introducing community-based mental health support in Serbia: A qualitative study on experiences and needs of long-term psychiatric users

**DOI:** 10.1017/gmh.2024.81

**Published:** 2024-10-17

**Authors:** Biljana Stanković, Petar Lukić, Irena Stojadinović, Jasmina Bogdanović, Maša Vukčević Marković

**Affiliations:** 1 Faculty of Philosophy, Department of Psychology, University of Belgrade, Serbia; 2 Faculty of Philosophy, LIRA Lab, University of Belgrade, Serbia; 3 Psychosocial Innovation Network, Belgrade, Serbia; 4 Institute of Mental Health, Belgrade, Serbia; 5 Faculty of Political Sciences, Department of Social Work, University of Belgrade, Serbia

**Keywords:** community-based mental health support, long-term psychiatric users, schizophrenia spectrum disorders, qualitative research

## Abstract

**Background:**

Mental health (MH) system in Serbia still relies heavily on the medical model with very restricted availability of community-based support. The aim of this study was to provide insight into the everyday experiences and unmet needs of psychiatric users suffering from schizophrenia spectrum disorders in Serbia who are also users of community MH services.

**Method:**

We recruited the participants (*N* = 11; 9 males; aged 26–65, *M* = 48.5), long-term psychiatric users (11–57 years, *M* = 29.4) diagnosed with a schizophrenia spectrum disorder, from a community MH centre. We conducted in-depth semi-structured interviews with them, which we analysed relying on the principles of thematic analysis.

**Results:**

Three broad themes relevant to participants’ well-being and quality of life were identified: leading a meaningful and fulfilled life; the importance of continuity of socialisation and support; and maintaining control and a sense of agency. Community MH services have markedly figured in facilitating all three dimensions.

**Conclusions:**

Findings suggest that providing continuous services that address the users’ need to engage in activities that give them meaning and purpose, provide socialisation and peer support, and promote their autonomy and agency can play a vital role in advancing the process of recovery and well-being of long-term psychiatric users.

## Impact statement

There has been a major re-examination of psychiatric treatment models in high-income countries influencing the transition to a recovery approach and a greater emphasis on broader accessibility of community-based mental health (MH) services. However, low and middle-income countries are often characterised by very restricted availability of community-based services and the persistent mental health treatment gap. Additionally, there is not enough evidence on psychiatric users’ perspectives and experiences in these countries needed to inform the adjustment of existing services, while previous studies suggest beneficial effects on recovery when services are in line with users’ preferences and needs. The recent initiatives towards the improvement of mental health care in Serbia and neighbouring countries suggest that it is of great importance to provide the perspectives of persons with mental disorders to inform community-based MH services tailored to users’ needs. In the local context where psychosocial interventions and community support remain limited and precarious, the present research provides crucial insights into the everyday experiences, resources and challenges of long-term psychiatric users. Findings identify key dimensions, outside disorder-related issues, impacting their well-being and quality of life, and highlighting the value of mental health services that provide structure, meaning and peer support. The analysis of contextual specificities and unmet needs informs practical recommendations that have the potential to influence policy and practice in both local and regional contexts – due to shared socio-economic circumstances and similar history of mental health provision and care. The potential impact of the study is also timely since recent policy advancements in Serbia announced an increase in the scope and availability of community-based mental health services.

## Introduction

In recent years, there has been a major re-examination of mental health (MH) practice and treatment of mental health conditions in communities worldwide in order to achieve the highest possible level of health and full participation in society, without stigma and discrimination (WHO, 2021). In high-income countries, these changes are reflected in the increased availability of community-based services, reduced prescription of medication, and more frequent use of psychosocial interventions and psychotherapy in the treatment of all mental disorders (Lehman et al., 2004; Zhou et al., 2018; WHO, 2021). On a conceptual level, this shift implies a transition from the traditional medical approach to mental health and illness, characterised by an exclusive focus on symptoms and disorders, to the recovery model approach. This novel approach focuses on building resilience, gaining control over MH problems, and having a meaningful life, despite the persistent symptoms of a disorder (Jacob, 2015). An increasing number of studies contributed to the validation of the recovery model (Loos et al., 2017; Slade, 2017), and suggested that the underestimated community resources, such as peer support, significantly contribute to better psychosocial treatment outcomes (Davidson et al., 2012; White et al., 2020) and that transition to community-based MH support model is related to the broader accessibility of MH services.

These developments are especially relevant for the improvement of the treatment of severe and chronic mental disorders such as schizophrenia spectrum disorders. There is evidence indicating that the transition to community-based services is associated with increased user satisfaction and quality of life in persons suffering from schizophrenia spectrum disorders, leading to the reduction in non-compliance with treatment, relapse, rehospitalisation, and the long-term use of psychiatric services (Thornicroft and Tansella, 2003; Killaspy, 2007; Coffey et al., 2019). Moreover, the level of disability caused by these disorders is very high and strongly impacts individuals and their functioning, as well as their families and communities (Caldas de Almeida and Killaspy, 2011), often leading to social isolation and exclusion. These can, however, be mitigated by the community mental health model of care (National Collaborating Centre for Mental Health, 2012).

There is an emerging body of evidence on the needs, perspectives, and everyday life of people suffering from schizophrenia spectrum disorders (Thompson et al., 2008; Vilà et al., 2016), including social relationships (Andersson et al., 2015; Palumbo et al., 2015), occupation (Argentzell et al., 2012; Doroud et al., 2015; Hancock et al., 2015), as well as the process of recovery (Doroud et al., 2021). It was shown that employment, but also occupational engagement such as hobbies, create meaning and hope, as well as structure and routine, thus being highly beneficial in the recovery process (Doroud et al., 2015; Hancock et al., 2015; Vilà et al., 2016). Engagement in everyday activities provides them with opportunities to learn about themselves, evaluate the impact of their illness, develop strategies to overcome barriers, and create meaningful and fulfilling lives (Doroud et al., 2021). Furthermore, the importance and positive effects of social support and social relationships were widely documented (Vilà et al., 2016; Piat et al., 2017). On the other hand, some of the main documented challenges in everyday functioning include insufficient activities to fulfil their time, a high degree of social isolation resulting in loneliness, and a high level of reliance on professional support for meaningful conversation and social contact (Hocking et al., 2005; Thompson et al., 2008; Milbourn et al., 2015; Vilà et al., 2016).

Even though most studies in question provided insights from high-income countries, and a significant lack of insight into the perspective and needs of service users from low- and middle-income (LMI) countries in Europe and globally is recognised (Saraceno et al., 2007; Rathod et al., 2017), there is an evident increase in global mental health literature and studies conducted in LMI countries. It is shown that the local contexts in these countries are still often characterised by the limited availability of community-based services and the persisting MH treatment gap (Patel et al., 2010; Semrau et al., 2019). Additionally, experiences of people suffering from schizophrenia spectrum disorders in these countries are often characterised by the lack of social contact and everyday activities outside of their homes as well as the high presence of stigma and discrimination in the community and within their family (De Souza and Coutinho, 2006; Chidarikire et al., 2021; Brooke-Sumner et al., 2024).

The studies from Serbia and neighbouring countries, in which outpatient psychiatric care is usually limited to prescribing medication (Ristić et al., 2020) and does not provide rehabilitation home or community-based services, were mainly focused on medical and pharmacological aspects of treatment and care (Ilić et al., 2010; Ćosović et al., 2016; Dutina and Stašević-Karličić, 2018; Jerotić and Marić, 2018; Javorac et al., 2019; Stašević et al., 2019). Although it was suggested that the social functioning and quality of life of people with schizophrenia need to be explored and understood to gain a clearer picture of the direction to be taken in treatment (Ristić and Batinić, 2020) and support the current development of community-based MH services (Šago et al., 2018; Repišti et al., 2022), these studies are virtually non-existent.^1^ A few studies from neighbouring countries of the former Yugoslavia that share a similar cultural context and health care system show that the quality of life of people with chronic mental disorders (i.e., schizophrenia spectrum disorders) is worse than the quality of life of chronic somatic patients, in areas of physical health, mental health, and social relations (Adrić and Babić, 2015), and that the most prominent unmet needs identified are related to financial and professional aspects of functioning, followed by needs related to health, unfulfilled everyday life and unmet need for love (Živanić-Stjepanović, 2017).

Therefore, both in Serbia as well as in other LMI countries, there is a lack of studies focused on understanding the perspective and experiences of psychiatric users, including people with schizophrenia spectrum disorders, which would be beneficial in further informing the development of community-based services in the context of Southeastern Europe.

### Challenges in implementing community-based mental health support in Serbia

The reform of the mental health care system in Serbia^2^ started in the early 2000s when significant advances were made at the level of national strategies and policies,^3^ advocating for the gradual transition from solely inpatient treatment to the development of community-based services. Unfortunately, the process of implementation of these policies is slow and hindered (Lecic-Tosevski et al., 2010). As a result, until recently, the system relied almost exclusively on specialised levels of care (inpatient care and pharmacotherapeutic treatment), and non-pharmacological interventions were rarely included in clinical practice (Stevović et al., 2022). However, the COVID-19 pandemic highlighted the importance of mental health protection, including community-based services (Torales et al., 2020; Nealon, 2021), which led to the reignition of the reform of the MH protection system in Serbia,^4^ and initiatives aiming to reduce the significant gap in the availability of community-based services.^5^ In addition, the first case of the school mass shooting that happened in May 2023 in Serbia put additional focus on the urgent need for increase in the availability of mental health services and introduction of newly available community-based services.^6^ Due to the above-mentioned local circumstances, it can be expected that over the following months and years, an increase in availability of community-based services will follow.

At the moment, in Serbia, there are few community-based MH centres within the healthcare system, as well as a few civil society organisations and user-led associations providing community-based services to psychiatric users. Within the healthcare system, there are six community-based mental health centres established within 20 years (from 2003 to 2023). Five of these centres are established by the hospitals specialised in the treatment of psychiatric disorders and are located in the same or nearby cities as these hospitals, and one centre is established within the primary healthcare unit. All centres provide clinic-based activities only. Some of them offer mainly individual psychological counselling services, whereas, in the others, the multidisciplinary teams are employed to provide different types of services, including psychosocial rehabilitation of those treated in the nearby hospitals. Thus, even though the centres are located in the communities, they are still closely linked to the institutions and the institutionalised model of care. In Belgrade, the capital of Serbia and its largest city, where about a quarter of the population lives, there is only one centre within the healthcare system that mainly provides individual psychological counselling and does not offer any community-based services for people with schizophrenia spectrum disorders. The only community-based services for people with this type of mental health difficulties in Belgrade are provided through the civil sector – “Association Prostor” which provides a multidisciplinary program of psychosocial support, and two user-led associations organising informal and self-help activities. “Association Prostor” is an organisation that provides community-based service to persons suffering from schizophrenia spectrum disorders for more than a decade. In November 2020, “Association Prostor” established a community-based centre for psychosocial support in Belgrade, offering a comprehensive psychosocial program named “Creative space for mental health” (hereafter: the Centre). The Centre is a unique type of mental health service in Serbia as it is the only community-based service for people suffering from schizophrenia spectrum disorders that offers a safe space for recovery in the community after the hospital treatment is finalised and provides a broad range of multidisciplinary services combining educational and occupational activities, group psychotherapy, art therapy, peer support, social welfare counselling, and socio-economic empowerment activities, such as exhibitions in the community and online podcasts.

However, what needs to be explored is whether these types of services are in accordance with the needs of users of psychiatric services, and the principles of the recovery model approach, since no previous study provided this type of evidence or services’ evaluation. Therefore, the main aim of the current study was to provide comprehensive insight into the everyday experiences, unmet needs, difficulties, and resources of psychiatric users suffering from schizophrenia spectrum disorders, and their experiences related to community-based services in the Centre. This insight would contribute to the improvement of these services as well as the development of the context-appropriate services that are yet to be introduced.

## Methods

### Sampling procedure

The participants were recruited from a community-based centre for psychosocial support (the Centre) run by “Association Prostor,” a civil society organisation in Belgrade, the only one in the country providing community-based services outside the healthcare system to persons suffering from schizophrenia spectrum disorders. The inclusion criteria for participation in the study were that the participants have been regularly using the services of the Centre (i.e., at least two times a month^7^), that they have a diagnosis of schizophrenia spectrum disorder, and a capacity to provide informed consent. We approached all current users of the centre that fit these criteria (15 users^8^) and those who wanted to participate gave their written informed consent and were involved in the study.

### Data collection

The first two authors conducted semi-structured individual interviews with the participants between March and July 2021 in the community MH centre in Belgrade. We asked participants to describe their typical day in detail (present or recent past) – their activities and practices, daily rhythm, responsibilities, free time, social encounters, and living conditions. We also asked them to describe one recent day that was especially good for them (that was fulfilled, when they felt happy and satisfied) and one that was especially bad for them (when they did not feel well or were particularly dissatisfied). We focused on the meanings they attribute to different situations and events, their interests and preferences, and their emotions and affects. Based on these detailed descriptions of experiences, we asked them to assess their main problems and difficulties, as well as their strengths and resources. Finally, we asked them to do an in-depth evaluation of and reflect on various aspects of the community-based services in the MH centre. Our aim when developing the interview agenda^9^ was to assess their various experiences, resources and challenges in detail and also to understand the role of community-based service in their everyday life. The development of the agenda was additionally led by two considerations, both connected to the cognitive and behavioural specificities of the participants: to keep the questions as concrete as possible and to keep the agenda relatively short. The interviews lasted approximately 45 min. Since this is a relatively short time to assess the participants’ life contexts in more detail, the relevant background information was provided by the third and fourth authors, who are very familiar with the participants, being regularly engaged with them as part of the community centre activities, since they have been working there as a psychologist and a social worker, respectively. Interviews were audio recorded and transcribed verbatim.^10^

### Ethical concerns

The participants were provided with information about the study and asked to give written consent for the interviewing and audio recording beforehand, and the interviews were scheduled to fit in with their other activities at the centre.^11^ At the beginning of the interview, the information about the study was explained again, including relevant ethical aspects (the issues of anonymity and privacy, the right to withdraw at any moment or skip some questions) and the participants were asked for their oral consent. The anonymity of the participants was ensured by using pseudonyms, and all identifying personal information was changed before the start of the analysis. All participants were offered the opportunity to receive debriefing and psychological counselling following the interview. Due to the lack of secured funding, participants were not compensated for their time.

### Data analysis

To analyse the data, we performed a thematic analysis (Braun and Clarke, 2006). The first two authors thoroughly familiarised themselves with the data to get a sense of the overall content and context of the material before starting the coding process in MAXQDA Analytics Pro 2020 software by selecting an especially rich subset of data and developing an initial list of codes inductively. All emerging dilemmas and disagreements were resolved through discussion and careful consideration of all alternatives. They continued the coding process of the remaining transcripts independently, starting from the initial coding scheme, and developing it further in accordance with the novelties in the material. All ideas for interpretations and for developing higher-order themes were continuously recorded by both researchers. They regularly compared the coding schemes and interpretations, resolved all inconsistencies, and together grouped the codes into themes. All transcripts were first analysed as case studies, before moving to cross-case analysis, based on which they reviewed and refined the themes and the coding scheme where necessary.

### Methodological integrity

We used several strategies to ensure the quality of the analysis by contributing to the validity and credibility of the findings. We developed the themes in an inductive and iterative manner, based on the case study analysis, so the contextual information and specificities of the personal stories could be preserved. The analysis was performed in a collaborative manner, which provided different perspectives on the interpretation of the data and enabled the consensus to be reached through critical reflection and discussion (researcher triangulation). The third, fourth, and fifth authors, who are involved in developing and provision of mental health and psychosocial support to psychiatric service users for years, provided additional background information on the participants and the local setting and discussed the findings in detail with the first two authors, which further contextualised and validated both the data and the analysis. The rigour of the analysis was promoted by using the QDA software, since it supports the consistency and transparency of the coding and analysis, and helps with documenting the whole process. Finally, to demonstrate that interpretations are grounded in the data, illustrative quotes from the interviews are provided throughout the Results section.

## Results

### Sociodemographic characteristics

Finally, 11 participants (9 males, 2 females) and aged 26–65 (*M* = 48.5) were involved in the study. They were all diagnosed with a schizophrenia spectrum disorder,^12^ having been regularly seeing a psychiatrist and taking prescribed medication. All of them have also been users of psychiatric services for years, even decades (11–57 years, *M* = 29.4), and have experienced multiple hospitalizations (1–15 hospitalizations, *M* = 7.0). Only one participant was employed at the time of interviewing, more than half of them were living with their parents, and the majority of them (70%) had a monthly income of less than $200.^13^ They had scarce social contacts outside the community centre services and most of them (73%) had not seen any friends in the week prior to the study. Sample characteristics are given in more detail in Table 1.

**Table 1.** Sample characteristics (*N* = 11)

	*N*	%
Age		
20–40	2	18%
40–60	7	64%
>60	2	18%
Educational level		
Incomplete high–school education	3	27%
High–school education	7	64%
Incomplete higher education	1	9%
Living conditions		
Living with parents/guardians	6	55%
Living alone or with a partner/children	5	45%
Employment		
Employed	1	9%
Unemployed^a^	8	73%
Retired	2	18%
Number of years using psychiatric services		
10–15	1	9%
15–30	7	64%
>30	3	27%
Number of hospitalizations		
0–5	5	45%
5–10	3	27%
>10	3	27%
Individual psychological support		
None	8	73%
Individual counselling or psychotherapy^b^	3	27%

a Three of them (25% of the sample) are proclaimed disabled and lost their work capacity, so they are not allowed to work.

b In the local context and according to the regulations in Serbia, counselling services (referring to psychological counselling) and psychotherapy services differ according to the education of the service provider. Psychological counselling is provided only by qualified psychologists, while psychotherapy is provided by professionals with different backgrounds who have completed training in psychotherapy. This difference in training leads to different knowledge and skills and thus to a different type of service being provided.

Although this is clearly a very underprivileged group, there is a reason to believe that they are actually in a better situation than most other long-term psychiatric users in Serbia since they all live in Belgrade, the capital city, and are involved in psychiatric users’ associations or and at least sometimes occasionally attend community support programs for years (between 2 and 11 years, *M* = 8.1). The socio-economic situation is less favourable and available resources are even scarcer in the rest of the country.

### Thematic analysis

**Figure 1.**
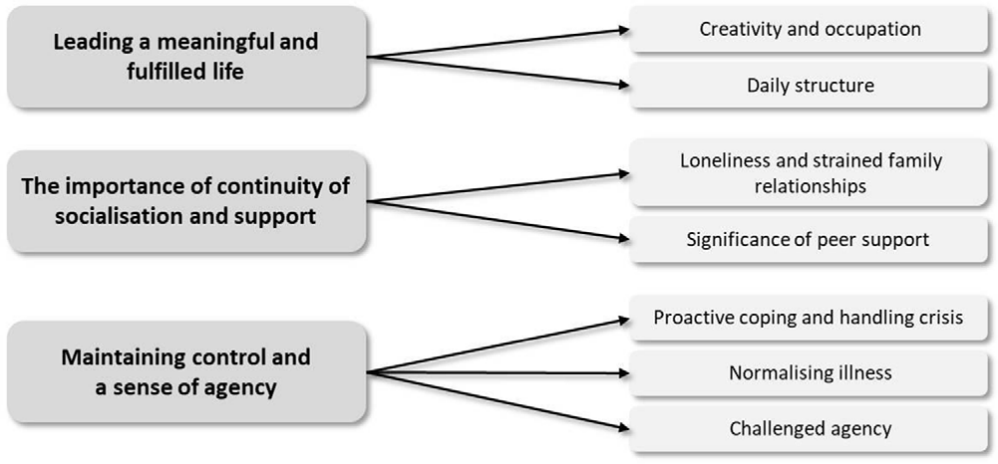
Overview of the main themes and subthemes.

We identified three key themes through the analysis (Figure 1), referring to the crucial dimensions of participant’s everyday experience, reflecting their needs and thus being closely related to how community-based MH services should be adequately organised to respond to these. These themes are (I) leading a meaningful and fulfilled life (as opposed to the lack of occupation and structure); (II) the importance of continuity of socialisation and support (as opposed to loneliness and lack of support); (III) and maintaining control and a sense of agency (as opposed to passivity and apathy).

#### Leading a meaningful and fulfilled life

An opportunity to lead a meaningful and fulfilled life is recognised as the main source of life satisfaction among the participants. The vast majority of participants have never worked or worked only in their youth before they were deprived of their work capacity, so the opportunity to be engaged and feel useful that the MH community centre offers them is of particular importance. Two subthemes emerged, both related to the organisation and content of MH support programs: creativity and occupation, and daily structure.

##### Creativity and occupation

The most frequently mentioned source of satisfaction is an opportunity to participate in purposeful activities and to be engaged. Most participants recognise creative activities primarily as opportunities for affirmation and improvement of their creative abilities and capacities (painting, poetry writing), which contributes to their sense of accomplishment and self-worth, and they feel pride for what they can achieve.

I am especially in a good mood when I can accomplish something, make something, contribute something. And then I’m so … excited, but in the normal range (laughter). Happy, let us say happy. (Nevena, 54)

Painting gives me one, how can I explain to you, the sense of security. And more confidence. Somehow, I’m happy when I can draw something and photograph it and send it to my son. (Vesna, 64)

When I worked with acrylic paints for the first time, I had never done oil on canvas before, if you could just see my expression… Forget about Rembrandt, I enjoyed it so much! (laughter) (Ilija, 51)

Besides creative efforts, activities with a pronounced manual component – sewing, handcrafts, computer repair, gardening – often help participants “divert thoughts” and relax, so they are not focused on negative content and can cope with their symptoms better (even very intrusive ones like hallucinations).

What’s more, in the moments when the madness swells up a bit, that work … I’ve tried it several times, to work with computers, and that work somehow puts me down … reduces the madness, it’s in the background, I still can hear something that is not there, I see something, some vortex on the wall, but it is not so intrusive, it is not so much… (Jovan, 65).

And when I have a problem, I like to work manually. (…) I constantly want to occupy my hands and when, for example, I crochet - I count and then I don’t think. That’s how I relieve my psyche and then I feel better. (Nevena, 54)

This implies that the emphasis on occupational activities as part of the MH support programs might bring psychological benefits (i.e., increasing wellbeing, improving coping mechanisms).

##### Daily structure

Even in the absence of some creative or productive activities, the minimum condition necessary to preserve the quality of life is that days have a predictable and organised structure – the usual sequence of everyday activities, daily routines and habits (sleeping, meals, taking medication, hygiene habits, watching a specific TV program, obligations, hobbies, exercising).

My average days are monotonous. So to speak. And they suit me. (…) I have no need for some… don’t look for some destabilisation of my monotonous life. That’s it. (Jovan, 65)

Others who do not have structured days find it difficult to talk about how they spend their time. Their narratives are poor in content and are dominated by negative feelings or apathy (passivity, lethargy).

Well, how I spend my days…I sleep a lot. (…) I don’t do anything special, let me tell you, uh (…) I mostly spend my days in bed. When it was winter, I had a hard time forcing myself to go outside at all. (Nemanja, 26)

(Exhale) What makes my day? What means something to me, you mean? (…) Yes… Well now, I don’t know what. (Long silence) I can’t answer you, I don’t know. (…) And I watch TV more than I should, but it seems that I am, uhm, well, I’m waiting for something like, I don’t even know. For my life to pass. (Mihajlo, 49)

The most indicative are the experiences of those users that are productive and creative when they participate in organised activities in the MH centre, but struggle to organise their days and find some occupation by themselves during the time they spend at home. They recognise those periods as especially challenging, since they spend time passively and their days often have an unclear or non-existent structure, which is why they highly appreciate routine obligations and activities offered from the outside.

So I go there (to the MH centre) to fill my day! Because I’m kind of empty if I don’t, I don’t go anywhere then. (Srđan, 50)

Well what am I going to do… I watch TV, look out the window when vehicles and people pass by and look at shops. (…) And after the weekend, gloomy and very difficult time over the weekend, Saturday and Sunday, we get together on Monday and everyone is happy. (…) Art therapy, or whatever it is called. Then we gather there and paint something. Coffee, juice and cookies, and so on … we are talking and hanging out. (Milan, 44)

This stark contrast between days or periods of time when they have and do not have activities or opportunities to socialise in the MH centre indicates the importance of continuous community support. This is especially true for those psychiatric users who have less internal resources to plan their free time and impose structure, or lack adequate social support otherwise.

#### The importance of continuity of socialisation and support

Most of the participants express a strong need for socialising and communicating, and at the same time emphasise problems in establishing and maintaining close and harmonious relationships with others. MH community support proves to be an important resource, especially by providing peer support.

##### Loneliness and strained family relationships as main challenges

Especially for participants who live alone, loneliness is a significant source of negative feelings and difficulties, so the need for company and communication is particularly strong – even contact with strangers or the company of pets is perceived as comforting.

Fear. I am even afraid of myself. (…) I don’t know why and how. When a person lives alone, hundreds of pieces of information, events, and different things cross his mind. I could hardly wait to come here to have this interview because I couldn’t handle it anymore. (…) Loneliness is hard for me. (Milutin, 53)

And then I was without a pet for a long time and it was harder for me than now with parrots. And uh … I’m happy now. (…) Because they have a soul as well, and then I am not alone. They have a soul… and then I talk to them when I’m not feeling well. When I’m down I talk to them and… Because when I’m alone, I get a little lost. My thoughts wander, and it’s not really … (Igor, 37)

For persons who have problems with internal organisation, loneliness contributes to feelings of disorientation and insecurity. A continuous relationship with others is therefore necessary in order to maintain not only their well-being but a basic experience of stability and continuity. When feeling unwell, many users actively seek social support as a way of coping – often by contacting a friend or a volunteer from the MH centre.

And a call from someone can improve my state when I’m feeling unwell… Or I call my friend Milan to see what’s happening with him… how is he handling it. (…) Or I call a volunteer or a psychologist and so on if something bothers me. (Srđan, 50)

However, the mere presence of other people or contact with them does not suffice, since the quality of relationships is what is important. For those participants living with their families, instead of loneliness, conflicting family relationships are very often a reason for dissatisfaction, frustration, and suffering. This is exacerbated by the specificity of their position - despite being adults, they are under the care of others, so they remain in the liminal position of persons who are certainly not children, but are not full-fledged adults either. They are often denied autonomy and therefore desire to emancipate and live alone, and the few who manage to do so derive great satisfaction from it.

Now I’m going to test myself to see how tolerant I can be with my father, cause he is a little grumpy. To see how I will deal with him when he tells me “take this, take that”. I need to practise that a little bit. And to be less afraid of his insolent behaviour or when he repeats constantly some things I already know and … treats me like a child. And then he’s like “you’re an adult, and you don’t know, and you are 50 years old”. (Srđan, 50)

You know what, I haven’t had any inconveniences since I started living alone. (…) If you could film there one day (while I was living with my family), that would have been… (…) There was constant drilling. When I moved away I first lay down for ten days. I used to be constantly on my feet, I ate standing up. It was such a pressing in the house that this period now is fantastic. (Ilija, 51)

Therefore, MH support programs may satisfy different needs of psychiatric users, depending on their varied family/life circumstances. For those users living alone MH support programs are the source of social relationships and support, as they can make friends or even meet romantic partners. For those who live with their families, engaging with the MH centre offers an opportunity to be involved in activities that are not supervised by their parents or legal guardians, thus practising autonomy they lack, as well as establishing egalitarian relations.

##### Significance of peer support

Peer support is often emphasised as particularly important. Other psychiatric users are recognised as significant companions and a source of support, since they are the only ones who can really understand what a person is going through because they have similar problems themselves. This makes it easier for a person to be open and authentic in communication, but also to learn to accept and normalise their condition through comparison with others and their experiences. That is why the availability of user associations that enable regular meetings and exchanges within this community is particularly important.

Since people come to this centre…with psychiatric problems. So, first of all, I can be relaxed. All the people I know outside the institutions know that I am a psychiatric patient, but still… I can’t tell them… it would be a burden for them if I told them what I hallucinated and this and that. And here, for example, we can talk quite freely about that. And it pleases me (…) That I can be comfortable. I can talk about … about what I experienced, what I saw, heard. And I can listen to others. (…) And it can help me not only to understand that person but also to understand myself and to learn something. (Jovan, 65)

The users recognised that regardless of the organised activities and services, it would mean a lot to them to have the MH centre available throughout the day, as a shared space for gathering, socialising, and mutual support, especially in situations of crisis when usually the only option available is hospitalisation.

It would mean to me if, for example, this space could be open for us, not necessarily twenty-four hours a day, but from eight to eight. And then I would stay longer and I would come more often… Even when there is no activity. To have some coffee and talk with others. (Milutin, 53)

There should be some kind of facility, a house or something similar, where one could spend the night, if someone is unwell and in need of support, but not so unwell as to go to the hospital, they should be offered support. (…) If there could be such a centre where the users would offer this to each other, not necessarily volunteers… (Ilija, 51)

These findings highlight the relevance of peer support in providing MH care in the local context. However, as this type of support is not formally recognised within the local system of mental health care, it is scarcely available and provided only by the civil society sector (and thus with limited sustainability, at least while without systemic support).

#### Maintaining control and a sense of agency

Most psychiatric users find it difficult to establish and maintain control and a sense of agency in different life spheres and aspects of their functioning – from their health condition and symptoms to major life projects such as finding a job or a partner. However, there are also strategies many participants use to establish control and experience agency, and MH community support could be beneficial in strengthening and promoting those.

##### Proactive coping and handling crises

Proactive attitude and willingness to take responsibility and initiative, especially when facing challenging situations, is emphasised as a significant source of self-satisfaction and an important coping strategy. Programs at the community centre can provide valuable opportunities for psychiatric users to practise and improve their skills and competencies, thus contributing to their self-confidence and proactive attitude.

Well, I’m satisfied with myself. I am trying. I think I’m trying. (…) Um … it’s kind of easy for me, when I fight, it’s easy for me. And, I paint at home and I listen … I have parrots, and… when I fight against bad thoughts, then I succeed. (Igor, 37)

We learned computers recently (as part of activities in the centre), so Milan and I, you know, as comrades, we got to grips with it, we fought with all our strength, and we learned something, and now we know something new. (Srđan, 50)

One of the strategies commonly used among high-functioning participants is that of rationalising and embracing problems and challenges. They are accepted as a normal part of everyday functioning and they do not dominate participants’ experience of quality of life.

Sometimes I’m not … in the mood, but that’s the way it is, I mean life is made up of conflicts also, and there are some beautiful things… so that’s normal. (Igor, 37)

You know, in a way, a lot of things bother me, but that doesn’t mean something is preventing me from functioning in life. (…) It will be difficult sometimes, but life is a very beautiful thing. (Ilija, 51)

Similarly, when it comes to handling symptoms or a worsened state, coping sometimes means leaving room to “feel ill” and leaving enough time and space to overcome the crisis.

Well, when I’m not well… let’s say, I languish a little. I sit for a while, I want to be alone so that no one bothers me. It’s just as if I’m gathering energy with myself and the strength to move on … and then it doesn’t last long. Maybe not even one day. That’s when I let myself, so to speak, to get ill, to suffer a little bit, then I put together something in my head, by myself, and I say: “Come on, let’s move on.” (Nevena, 54)

Coping with acute symptoms sometimes implies a high level of reflectivity and self-control. One long-term psychiatric user describes how he learned to recognise signs of an approaching psychotic episode, which allows him to prepare and isolate himself, to give himself the space to “go crazy”.

When it comes to that fuss, which is called psychosis, schizophrenia, or insanity, I have this one specific characteristic that makes it easier for me. It has evolved over time. First I hear some ringing, because I know it’s not … in the beginning, I checked the phone, however, it was a long time ago, I realised that it is of course a hallucination. And that bell informs me that something very awkward, much stranger is approaching. Well, then I just leave all obligations. And I lock up, and then, uh, I let myself go crazy. Yes, I see, I hear things that only I can see and hear. (laughter) And that makes me pretty dysfunctional. Then I don’t even answer the phone. (Jovan, 65)

While these coping strategies might not be useful or viable for all psychiatric users, the opportunity to talk about them openly, exchange experiences and validate and encourage different ways of handling crisis should be included in community support services.

Finally, when it comes to straightening or developing coping strategies that could help psychiatric users handle their symptoms, an important unmet need is access to psychotherapy or psychological counselling, since these are the MH services commonly unavailable to psychiatric users in Serbia.

Well, I would introduce some kind of psychological support. But real psychological support. (…) Like psychotherapy, yes. It would mean a lot. Yes, that would mean a lot to me. (Vesna, 64)

What I really miss…is individual psychotherapy. Would it be possible somehow to organise it in the Centre, for example, to determine one day for it, I don’t know, and to make a list (laughter) of who will do it when, on an individual basis, for example, if someone needs it. Because I really can’t pay for this. Financially, I can’t handle it… Because when I go to the psychiatrist for a check-up, it’s ok, I also tell how I feel physically, mentally, and emotionally and what troubles me, finances and this and that, but in the end again, hmm … I often can’t get that from my doctor and I really miss that understanding, so to speak. Or, as they say, feedback. (Nevena, 54).

##### Normalising illness

Acceptance and normalisation of one’s condition and diagnosis appear as particularly significant aspects of coping for psychiatric users. Some are identified with the assigned social identity (that of a patient) and over time they have become familiar with the specificities of their condition and have learned to react to it.

Since I am a psychiatric patient…I don’t like this service user – a psychiatric patient. (…) Even when I go crazy, I’m not bad. Unlike a lot of people who are afraid of their madness, I’m not afraid of my madness, I know it. (Jovan, 65)

However, accepting the medical framework does not necessarily exclude alternative discourses (e.g., spirituality, parapsychology) that offer a different interpretative framework for someone’s experiences. Even though it is recognised that such discourses are treated as unwarranted or even symptomatic by the medical establishment.

I’m very interested in this… underrated area that is even called parapsychology… I’m quite seriously interested because I often had some and… and now I have some… some experiences that I’m sure are not madness… (…) But, um, I don’t talk often about it, never with doctors. (laughter) Well, no, those people, what should they do. They are professionally used to having messiahs come to them. (…) They immediately classify it as psychosis. It makes no sense! And I don’t want them to increase my therapy. (Jovan, 65)

Importantly, some artistic techniques are recognised as tools for creatively representing the dynamics of fragmentation and integration that is central to psychotic symptomatology and for communicating this to others.

Someone calls it an installation. Two or three realistic figures are put together and placed on a stand, and then when they are illuminated with light, those three figures give a fourth realistic figure on the wall, in the shadow. What does it mean? Tribe F20^14^ is a tribe of schizophrenics. (…) Well, the whole concept is actually that we want to show that we have split into several personalities, into those figures…and that looks scattered…but when we illuminate it, we get a real figure again. So we are trying to recompose. We are not the same as we were. We are trying to assemble ourselves in some new way, in some form, to be whole again. (Nevena, 54)

Artistic techniques such as the one above represent an important part of existing MH community services in the centre. Actually, most participants emphasise the significance of art therapy for them, which, apart from giving them the possibility to be creative, allows them to express themselves, as well as to get to know themselves and other users through talking about what they have drawn or painted.

Art therapy suits me. Well, there you express, I guess, how you feel and all that. (…) And you draw, everyone draws, and then everyone presents their work and then we discuss it. It’s interesting because you can ask whatever interests you, to learn how other people feel, how you feel and so on. (…) This also relaxes me, it benefits me, in a way I also get to know myself, if nothing else. (…) To me, that drawing is a bit like a kind of distorted mirror. It doesn’t have to be a literal reflection, but it speaks of me, so I like it. (Mihajlo, 49)

I drew a picture (in art therapy). And I was explaining to her (the art therapist) what this is, what this is. I drew this because this is how I feel, I drew this because I feel this way. And she understands it well, says ok, and then gives you some advice and the advice is always good. (Milutin, 53)

These findings highlight the importance of including diverse resources for creatively expressing, exploring, normalising and communicating MH states and problems, since these are recognised as significant coping strategies among the participants.

##### Challenged agency: The lack of material resources

Main challenges that stand in the way of more active involvement and a sense of agency are connected to the lack of resources, primarily financial security and the possibility to get a job. Many participants report a poor financial situation and economic uncertainty as a significant source of difficulty. Given a very limited or non-existent possibility of employment (most were deprived of working capacity at a young age), psychiatric users rely on family pensions, financial support of family members, or social welfare. This life sphere is the one they would most often like to change or improve (besides intimate relationships), but they do not see any concrete way to achieve that.

My finances are bothering me, I don’t know, ever since I moved to Belgrade (…) So finances are a problem for me and it is getting worse for me and my mother. We don’t have enough money, to buy cigarettes, to buy food and drink, to pay the bills… for a mobile phone, for a TV… It wasn’t this bad before, now it is difficult to earn something. By the way, the only thing I do in Belgrade is Liceulice (selling street paper). If there was no Liceulice, I don’t even know what I would do… (Milan, 44)

Well, it’s hard for me that I don’t have any money now. I’ve been without social benefits for a year now. (…) And then I had a hard time and cadged cigarettes… my grandmother gives me money, but it’s temporary, I mean I’m ashamed to take it from her. She gives because she is sorry for me. But I have to get back my social benefits… so that I feel better, and then all my friends are better, and women are better when I have money… and everything is better, even animals (laughter). (Igor, 37)

This participant lost his social benefits because he was struggling with bureaucracy and could not handle the complex legal procedures by himself. This indicates the need for a wider range of support services that would also include social services in the community, specifically focused on providing support in solving the financial, work and housing problems, which is particularly significant in a context where long-term psychiatric users represent a materially and socially vulnerable group.

## Discussion

This study aimed to gain insight into the experiences, challenges and resources of psychiatric and MH community program users in Serbia in order to inform the tailoring of community MH services to their needs. Despite various differences in life circumstances, current condition, and internal and external resources among the participants, three key dimensions of experience relevant for all users were identified: the opportunity to live a fulfilling and productive life in spite of the challenges they may face; the availability of harmonious close relationships and sufficient social support; and the possibility to maintain their agency and self-control by coping proactively with various difficulties.

These findings are in line with most of the international literature on the subject. Previous studies also indicated that occupation and productivity provided both structure and a sense of fulfilment, belonging, empowerment, and self-validation to psychiatric users (Thompson et al., 2008; Kinn et al., 2011; Doroud et al., 2015; Hancock et al., 2015; Vilà et al., 2016). What was especially emphasised in our study is the role of creative and manual activities as resources in symptom management since focusing on some practical activity serves as a coping mechanism that helps some users avoid being overwhelmed by symptoms, even when these are intense, such as hallucinations.

While participants living alone often stressed loneliness as a source of negative feelings and even threatening to their well-being and sense of self, those living with their families emphasised their lack of autonomy and strained family relationships. Both point to the importance of continuity of socialisation and support for psychiatric users, which was also widely documented in previous studies (Milbourn et al., 2015; Palumbo et al., 2015; Vilà et al., 2016), although the importance of having a space to be alone and autonomous when needed is also valued (Andersson et al., 2015). As suggested previously (Davidson et al., 2012; White et al., 2020), peer support is considered to be especially significant because participants feel they could be relaxed and authentic around people sharing similar concerns by not feeling awkward about their symptoms. Through exchanging experiences, the users learn about themselves, their illness, and possible coping mechanisms. Those who lack personal resources (capacity for reflection, adequate emotional regulation, and self-control) and/or adequate support from their surroundings could especially benefit from this by gaining a better understanding of their condition and feeling more in control of their symptoms and reactions in times of crisis. The possibility of having continuous (peer) support available especially in situations of crisis is recognised as a means of preventing hospitalisation in some cases.

Facilitating various coping strategies through MH community support programs that could help users maintain wellbeing and a sense of agency is especially important. Available services could be further expanded to the development of tailored programs for the time spent between two visits to the MH centre (i.e., helping with imposing daily structure, adopting hobbies, solving practical problems, etc.). Additionally, individual psychotherapy or psychological counselling is recognised as an important unmet need in the existing MH support programs, especially since it is usually unavailable to Serbian psychiatric users otherwise due to the lack of finances. Religious and spiritual beliefs and practices were not often emphasised, but for some users were very important as coping strategies. These are typically dismissed inside the psychiatric discourse and interpreted as symptomatic (Cohen, 2008), but the community support service providers could be more open and sensitive when it comes to the personalised meaning-making processes, even when those diverge from the dominant discourses. Supporting ways of thinking and dealing with symptoms outside the medical model, particularly through art, could be especially helpful.

Psychiatric users in Serbia generally belong to an extremely underprivileged part of the population and face a lack of resources and systemic support, similar to the situation in many other countries. For many users, a poor financial situation, unemployment (and a seriously limited possibility of getting a job), and economic uncertainty represent a significant source of anxiety and difficulties. This unfavourable situation limits their agency in many spheres of everyday life due to the lack of resources and a dependent position inside the family. This is in line with the finding of the study from Bosnia and Herzegovina, where most of the participants emphasised the lack of finances and employment as the main unmet need (Živanić-Stjepanović, 2017). The fact that it is extremely hard for many psychiatric users to change their financial and occupational situation under existing broader social circumstances makes any kind of financial and occupational support of crucial importance. Finally, considering this unenviable socio-economic situation of most psychiatric users, it is crucial that community-support programs are provided free of charge and thus broadly accessible. Current community-based services are free but operate in limited capacity and locations and with questionable sustainability.

Participants in this study, thus, highlighted how their personal recovery journeys involved the need to engage in meaningful activities and to maintain their sense of agency. This finding corroborates the assumptions of the recovery approach that provides people diagnosed with severe mental disorders with the opportunity to become decision-makers within their recovery process and take back control over their lives, while also underlying the fact that engaging in daily activities is a way of providing meaning and a sense of personal agency (Borg and Davidson, 2008; Piat et al., 2017; Doroud et al., 2021). While there are multiple obstacles to implementing this approach into the healthcare system in Serbia due to the lack of financial, organisational, and professional resources within institutions (Jarić and Milenković, 2017; Stevović et al., 2022), broadly available community-based support services organised and funded through cross-sectoral and public–civil cooperation could be a more sustainable alternative.

While this study offered valuable insights, there are some limitations regarding its sample and methodological approach. The study is relatively small due to the specific characteristics of our target population - individuals with schizophrenia spectrum disorders who are long term psychiatric users, but also have experience with community-based services operating outside of healthcare institutions. The fact that there is only one such community-based MH centre in Serbia reduced both the size and heterogeneity of our sample, especially in terms of age and gender. Additionally, participants in this study are underprivileged in many ways, but they all live in the capital city and have access to community support programs. Namely, most of the Serbian psychiatric users’ socio-economic situation and available resources are even scarcer, especially if they are also vulnerable and marginalised in other ways (e.g., living in rural areas, being extremely poor, elderly, seriously ill, etc.). Future research should thus include a more diverse sample of participants – and especially those very under-privileged and involved only in psychiatric and inpatient treatment. This would broaden the scope of the target population and thus allow for a larger number of participants to be sampled - contributing to the validity of the research findings and allowing potentially specific needs of different age, gender and social groups to be assessed. The methodological approach should also be diversified, particularly in the direction of innovative and art-based research (using photography, drawing, or painting as a means of expressing and documenting), as already suggested in the literature (Noiriel et al., 2020). Since it is challenging to study the experiences of persons who often struggle with verbalization, using only traditional qualitative research methods that rely heavily on verbal communication might offer limited insights. Additionally, a longitudinal study design can be useful in examining the recovery process and broader long-term effects of MH programs and interventions in a contextual manner, and without overburdening the participants.

## Conclusion and recommendations

Long-term psychiatric service users remain an extremely underprivileged and socially invisible group in the Serbian context, facing a very low quality of life because of poverty, social exclusion, stigmatisation, and lack of support. Although many advances are made at the level of national strategies and policies, the mental health care system is still almost exclusively based on in-patient treatment and pharmacotherapy, with a limited number and restricted availability of community MH support options. Qualitative analysis of lived experiences and needs of long-term psychiatric users shows that MH community support services promote and enhance their well-being and quality of life. This is achieved by providing assistance, guidance, and resources (both professional and material) to help them lead engaged and meaningful lives despite the challenges, by offering social support (especially from peers), and by backing up their self-management and coping strategies. On the other hand, the most important unmet needs point to the ways of improving and expanding existing MH community services in Serbia and other similar contexts by:

- *Providing stable and continuous MH support* through establishing and sustaining community MH centres, instead of occasional (and precarious) short-term MH support programs.
- *Promoting users’ autonomy, engagement, and establishment of egalitarian relationships* instead of paternalising and asymmetrical ones through users’ active involvement in proposing and tailoring the content within the MH support programs.
- *Providing regular and well-structured MH services* since that can help users build routines and habits, which is especially important for those who have fewer internal resources or lack adequate social support otherwise.
- *Diversifying MH support programs* to include: a) creative and educational activities that could affirm and improve users’ knowledge and skills, but also various occupational activities that facilitate better coping while also promoting inclusiveness since they are fit to users with various capacity and skill levels; b) various, especially art-based, resources for handling symptoms and crises; c) personalised services (sensitive to the strengths, difficulties, and life contexts of individual users – which is completely absent from their current institutional treatment and care) such as psychological counselling or psychotherapy.
- *Fostering peer support* through: a) recognising peer support as an important aspect of the provision of community MH care in the local context; b) providing constant access to a communal space of a community MH centre to enable continuous peer support (especially relevant in times of crisis to possibly prevent frequent hospitalizations); c) financially and institutionally supporting and promoting users’ associations to enable broadly available and sustainable peer support.
- *Providing practical assistance* through expanding social services in the community and multi-sector support to help users handle financial, work, and housing difficulties.

Therefore, providing community-based MH support programs in a sustainable, continual, and broadly available manner is especially important in under-resourced contexts, where they can play a vital role in promoting the recovery and well-being of long-term psychiatric users and help reduce the need for more institutionalised care.

## Data Availability

The interview data and coding manuals contained in this manuscript are not openly available due to privacy restrictions set forth by the Institutional ethics board but can be obtained from the corresponding author following the completion of a privacy and fair use agreement. The interview guide is made openly available at https://osf.io/wvrbu/?view_only=5eb937b15ba041cda58bf41c1055572f.

## Footnotes

1 To our knowledge, only one study dealt with the possibility of providing psychotherapy (specifically psychodrama) to people with psychotic disorders to improve their quality of life (Veljković, 2019).

2 Healthcare system in Serbia is publicly financed through mandatory healthcare insurance and provided through a network of medical institutions organised on three levels – university hospitals and clinical centres, general and specialised hospitals, and primary healthcare institutions.

3 First, the National Strategy for the Development of Mental Health Protection for the period 2007–2017 (Government of the Republic of Serbia, 2007) and then the Law on the Protection of Persons with Mental Disorders (Government of the Republic of Serbia, 2013) were adopted. These were followed by the National Program for Mental Health Protection for the period 2019–2026 and the Action Plan for its implementation (Government of the Republic of Serbia, 2019).

4 In July 2021, conclusions from the first national dialogue on mental health organised by the Ministry of Human and Minority Rights included the need for further improvement of community-based MH services (https://bfpe.org/u-fokusu/zastita-i-unapredjenje-prava-gradjana-u-oblasti-mentalnog-zdravlja-u-srbiji/).

5 Programs of promotion and prevention or psychological treatment rarely exist. At the primary health care level mainly psychiatric services are available, if any. At more specialised levels of healthcare, there is inpatient and outpatient treatment in general hospitals as well as in special hospitals for mental disorders and in clinical centres.

6 The government of the Republic of Serbia established a working group (WG) in May 2023 focused on mental health protection and safety of youth. The WG started a project run by the Institute of Mental Health focused on the introduction of new community-based mental health services in Serbia (https://www.dps.org.rs/2023/3846/).

7 Some programs in the Centre are scheduled on a 2 week period, that is, twice a month, and there are users who attend only these. So this criterion was chosen to include them in the sample.

8 The capacity of the Centre at the time of data collection did not allow for a substantially larger number of users to attend the programs. Additionally, the COVID-19 pandemic that closely preceded the period of data collection somewhat reduced the number of users.

9 Full interview agenda is available at https://osf.io/wvrbu/?view_only=5eb937b15ba041cda58bf41c1055572f.

10 Since the interviews were conducted in Serbian, the illustrative quotes in this article are translated by the first author (who is familiar with the data and thus with the context of the selected quotes) and then proofed and edited by an experienced English translator.

11 The third and fourth authors who were already involved in different activities with the participants in the MH centre described the study in detail in advance (aims, content and expected duration of the interviews, how the data will be used) and introduced the interviewers.

12 F.20.0 and F.20.5, with one participant being diagnosed with F29.

13 Which was less than a half of the current minimum wage in Serbia (https://wageindicator.org/salary/minimum-wage/serbia/archive/20210101).

14 An informal and self-organised (as well as self-financed) group of psychiatric users mostly devoted to creative and artistic work.
