# Supplementary material for: Introducing community-based mental health support in Serbia: A qualitative study on experiences and needs of long-term psychiatric users
Source: Glob Ment Health (Camb). 2024 Oct 17;11:e81. doi: 10.1017/gmh.2024.81 (PMC11505036; doi:10.1017/gmh.2024.81)
Supplement: Peer review history

# Editor report: Recommendation: Introducing community-based mental health support in Serbia: A qualitative study on experiences and needs of long-term psychiatric users — R0/PR2

**DOI:** 10.1017/gmh.2024.81.pr2

**Affiliations:** Director, National Institute of Mental Health, Czech Republic

No accompanying comment.

# Editor report: Decision: Introducing community-based mental health support in Serbia: A qualitative study on experiences and needs of long-term psychiatric users — R0/PR3

**DOI:** 10.1017/gmh.2024.81.pr3

**Affiliations:** London School of Hygiene & Tropical Medicine, United Kingdom of Great Britain and Northern Ireland

No accompanying comment.

# Editor report: Recommendation: Introducing community-based mental health support in Serbia: A qualitative study on experiences and needs of long-term psychiatric users — R1/PR5

**DOI:** 10.1017/gmh.2024.81.pr5

**Affiliations:** Director, National Institute of Mental Health, Czech Republic

No accompanying comment.

# Editor report: Decision: Introducing community-based mental health support in Serbia: A qualitative study on experiences and needs of long-term psychiatric users — R1/PR6

**DOI:** 10.1017/gmh.2024.81.pr6

**Affiliations:** London School of Hygiene & Tropical Medicine, United Kingdom of Great Britain and Northern Ireland

No accompanying comment.
